# The Rise of the Empowered Physician in the Digital Health Era: Viewpoint

**DOI:** 10.2196/12490

**Published:** 2019-03-26

**Authors:** Bertalan Mesko, Zsuzsa Győrffy

**Affiliations:** 1 The Medical Futurist Institute Budapest Hungary; 2 Department of Behavioral Sciences Semmelweis University Budapest Hungary

**Keywords:** e-physician, e-patient, empowerment, doctor-patient relationship, digital health, technology

## Abstract

Being a 21st-century health care provider is extremely demanding. The growing number of chronic diseases, lack of medical workforce, increasing amounts of administrative tasks, the cost of medical treatment, and rising life expectancy result in an immense challenge for medical professionals. This transformation has been triggered by the growing presence of digital health. Digital health does not only refer to technological transformation; it also fundamentally reshapes the physician-patient relationship and treatment circumstances. We argue that patient empowerment, the spread of digital health, the biopsychosocial-digital approach, and the disappearance of the ivory tower of medicine lead to a new role for physicians. Digital health allows the job of being a medical professional to become more rewarding and creative. The characteristics of a physician-as-idol could shift from self-confident to curious, from rule follower to creative, and from lone hero to team worker. Empowered physicians (e-physicians) can be described as “electronic,” where they use digital technologies in their practice with ease; “enabled,” where they are enabled by regulations and guidelines; and “empowered,” where they are empowered by technologies that support their job and their empowered patients (e-patients). They can be described as “experts” in the use of technologies in their practice or in knowing the best, most reliable, and trustworthy digital health sources and technologies. They can also be described as “engaged,” when understanding the feelings and points of view of their patients, giving relevant feedback, and involving them throughout the whole healing process. The skills and approaches that characterize this era of e-physicians, such as face-to-face communication skills, digital literacy, interdisciplinarity, knowing where to find information, translating large amounts of data into insights for patients, among others, should always have been at the core of practicing medicine. However, the economical, technological, and administrative burden of the profession has not made it possible for most physicians to enjoy the benefits of their training, individual capabilities, and creativity. By understanding how digital health technologies can support or augment their capabilities, physicians would have the chance to practice the art of medicine like never before.

## Introduction

The 21st century has brought unprecedented challenges to medical professionals. The growing number of chronic diseases, global doctor shortages, increasing costs of medical treatment, and rising life expectancy all together result in an immense challenge for medical professionals [1]. Managing and treating the increasing dominancy of chronic conditions started to depend more on cooperation between physicians and patients than on individual decisions. Physicians are pressed to integrate health IT into their jobs, while also trying to stay up-to-date with emerging technologies. They often have to deal with low-quality, bugged, or inefficient software and technologies that further decrease the amount of time they can spend with their patients [2].

Every second physician suffers from burnout according to a study by the American Medical Association; several other studies have highlighted the same observation worldwide [3,4]. The four major causes of burnout are bureaucratic tasks, spending too many hours at work, feeling like a cog in the wheel, and increasing computerization of practice. Additionally, having to balance between medically justified, economically affordable, and morally acceptable solutions is the trilemma of modern medicine. Therefore, being a 21st-century health care provider is extremely demanding.

The era of digital health, a cultural transformation that brings disruptive technologies to both patients and health care providers, might so far have benefited patients more than physicians. Physicians are trained to act as demigods who should not say, “I don’t know,” and should have all the information at their disposal, even when there are over 28 million medical papers in the PubMed database. In the meantime, information, knowledge, and technologies within the ivory tower have started to become accessible for anyone through new digital tools, social media, or crowdsourcing.

As such fundamental changes appeared only in a matter of years, while neither regulations, medical education, nor guidelines followed them, a fight-or-flight reaction has become common among physicians. Thus, many of them are either reluctant to adopt digital health or they protect the power they are used to having [5]. Not only has the sheer amount of information grown, it has also become crucial to know and be able to use even the latest technologies from apps and telemedicine to health sensors and portable diagnostic devices.

The new phenomenon we call *digital health* has initiated changes in providing care and in practicing medicine. Digital health is defined as “the cultural transformation of how disruptive technologies that provide digital and objective data accessible to both health care providers and patients leads to an equal-level doctor-patient relationship with shared decision-making and the democratization of care.” As technological innovations become inseparable from health care, and as health care systems worldwide are becoming financially unsustainable, a paradigm shift is imminent [1]. The cultural component of this transformation implies that how the stakeholders of health care adopt or reject new technologies is more dependent on the outcomes than how the technologies progress.

In summary, using digital technology has become unavoidable in practicing medicine, and empowered patients, also known as e-patients, have needs that are different than what medical education prepares physicians for. Today’s physicians are looking for their place, authority, and function in this status quo.

## Patient Empowerment Has Been Booming

In the second half of the 20th century, the biomedical model of medicine has been replaced by the biopsychosocial paradigm [6]. The biomedical model states that the biological determinants are the main causes of diseases. On the other hand, in the third and fourth epidemiological periods, it has become obvious that psychological and social elements of disease are equally important in development of diseases. We should not only seek one cause, but also the complex interaction of predictors, triggers, and maintaining factors.

Later, this approach has been complemented by a digital component, thus making it the biopsychosocial-digital model. The digital component means that the digital expansion of the biological self, the engagement of technology, and the use of online networks are as notable as the other biopsychosocial factors [7].

The digital component could affect health outcomes in many ways. For example, portable devices support management of health and enable affordable access to people with low socioeconomic status and/or in remote environments [8-12]. It was shown in a systematic review that technology could also be used to reduce the disparity in melanoma incidence, mortality rates, and accessibility to posttreatment care management between urban and rural or remote populations [13]. Online social networking has a potential effect on health, for example, through social support; also, interactive information-sharing has an influence on patient health and health behavior.

The story of how Dave deBronkart—otherwise known as e-Patient Dave—used technologies to help in the treatment of his cancer shows the contribution patients can make to the complexities of medicine [14].

Moreover, in the 21st century, personalized medicine has become unavoidable in treating certain conditions, such as several types of cancer or diabetes [15,16]. The challenge is that physicians are required to come up with solutions tailored to each patient’s needs instead of using treatment pathways of mass production.

Not only were physicians affected by the advent of the Internet and new technologies, but these technologies have also reshaped the lives and disease management of patients from the ground up. The e-patient movement came to life by raising issues and challenges that medical curricula do not address. The patient’s reaction to changes in access to information is understandably to participate in the healing process [17].

E-patients are active in their care and demonstrate the power of the participatory medicine model. The “e” can stand for “electronic” (ie, uses digital technologies in their disease or health management), “equipped” (ie, has digital health technologies at their disposal), “enabled” (ie, has newly acquired access to information), “empowered” (ie, by the loss of the ivory tower), “engaged” (ie, taking an active part in their care), and “expert” (ie, in using technologies in their care or health management) [14,18].

We argue that patient empowerment, the spread of digital health, the biopsychosocial-digital approach, and the disappearance of the ivory tower of medicine lead to a new role for physicians. Instead of key holders to the ivory tower of medicine, they are slowly transforming into guides for their patients in the jungle of health care and digital information.

We also need to emphasize that knowledge can only potentially mean power. Therefore, it will definitely be challenging for medical professionals to adapt to not being an intermediary (ie, someone who consumes information and passes it on), but to become an apomediary (ie, someone who directs the patient to high-quality information and services) and, thus, stop being a prerequisite to obtaining information. This new approach also means that patients will not be labelled as such, but will be labelled as consumers, users, citizens, or persons who may already use the public resources of digital health [19,20].

We also propose that it is time to empower physicians in the same way patients have been empowered and to let them use their unique vision, knowledge, and insights to help make the best decisions for patients aided, not replaced, by advanced technologies. Thus, the era of digital health not only means to equip e-patients with information, tools, and technologies, but also to equip empowered physicians (e-physicians) with time, opportunity, and technologies to fulfil the modern vision of a practicing physician. Here we discuss the potential ways of facilitating this transition.

E-patients have become experts about their illnesses, while their chosen health care providers help them to be able to help themselves. This has sparked new expectations from patients, from monitoring and recording to sharing their data. As practicing medicine becomes a collaborative process, not only among health care professionals but also involving patients, the features of both e-patients and e-physicians become comparable.

The “e” in e-physicians can also stand for “electronic“ (ie, use digital technologies in their practice with ease), ”equipped“ (ie, have digital health technologies at their disposal), ”enabled“ (ie, by regulations and guidelines) [21,22], ”empowered“ (ie, by technologies that support their jobs and their e-patients), “engaged” (ie, need compassion and empathy to understand the feelings and points of view of patients, give relevant feedback, and involve them throughout the whole healing process), and ”expert“ (ie, in using technologies in their practice or know the best, most reliable, and trustworthy digital health sources and technologies) (see Table 1).

The doctor-patient relationship has been changing due to digital technology and the shared access to information. Insights about medical issues and the use of technology can now come from both sides. They are moving toward shared decision-making, communicating extensively, and managing health and disease through teamwork.

**Figure 1:**
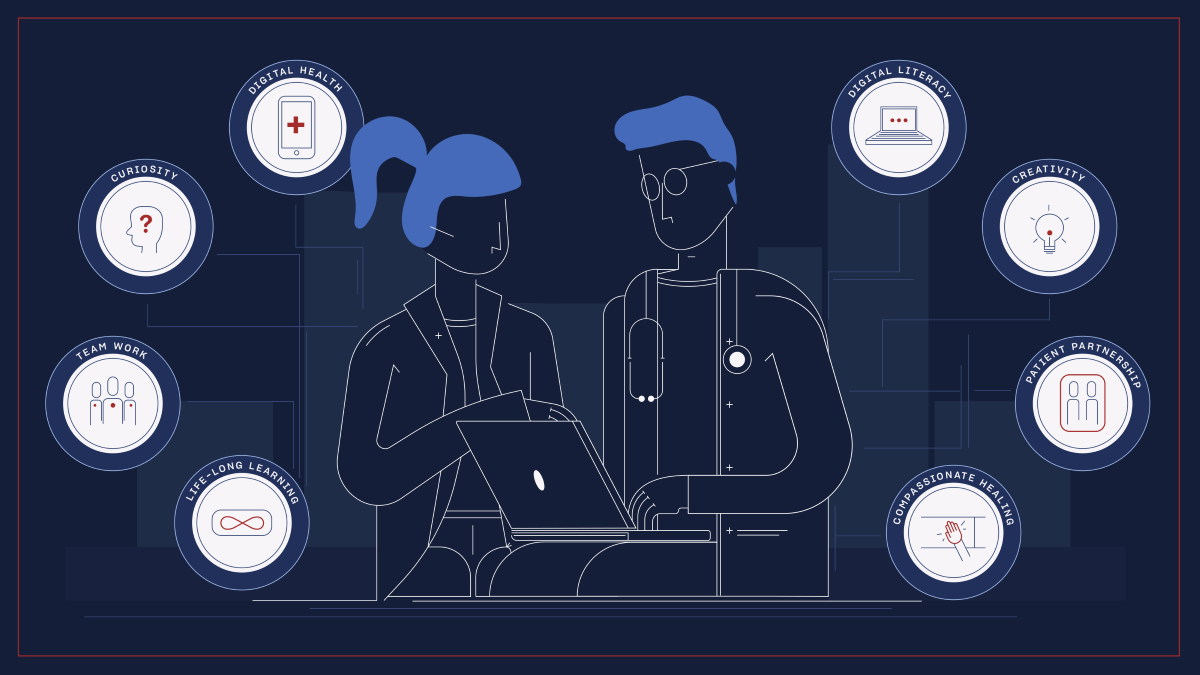
Schematic view of the approaches, skills, and features of an empowered physician (e-physician).

Digital health further offers the opportunity to make the job of being a medical professional rewarding and creative. While advanced technologies such as narrow and general artificial intelligence might seem to threaten replacement of physicians, they are more likely to support them and reduce the repetitive elements of their job that do not require the attention of a human mind. Thus, by adapting to the cultural changes initiated by digital health technologies, the characteristics of a physician-as-idol could shift from self-confident to curious, from rule follower to creative, and from lone hero to team worker (see Figure 1).

Certain skills are therefore crucial for e-physicians of the 21st century. Since there are more and more elderly patients struggling with chronic and polymorbid diseases, health care providers should be able to form an appropriate relationship with patients. Sufficient communication skills and the involvement of patients in prevention and treatment will become more important than ever. Adapting to constantly developing technologies is necessary and clinical skills should be improved with that in mind. The location of care has moved to the patient’s personal space (ie, home), which has been made possible by monitoring from afar with wearable sensors and portable diagnostic devices, among other technologies.

With the headway of telemedicine, new skills are needed regarding how to diagnose a patient and communicate with them without first a personal contact. Health care providers will need to be trained in such a way that they can diagnose, treat, educate, and monitor patients who are far away. A further improvement of this is the *hospital at home program*, which can allow more complicated treatments (eg, dialysis) to be available in the patient’s home, thus lowering the costs of hospital care [23].

Efficient teamwork is indispensable, since the development of science and technology makes it practically impossible for a healer to solve all challenges of a case on their own. As knowledge and treatment become more globalized, international research teams and the ability to work with clinical teams will be necessary [24].

Technology-focused professionals are also becoming a part of the team. New health care-related professions are going to emerge, such as clinical data scientists, medical software engineers, or digital medicine specialists [25].

The skills of managing, protecting, and orienting within datasets will also become irreplaceable. An e-physician will need to handle the information at hand in a critical and selective manner. The 21st-century healer will have to realize the ethical challenges created by digital health. For example, the way health-related data is collected, stored, accessed, and shared is an enormous privacy issue [24].

There are major factors that facilitate the transition of physicians from demigods to guides who enjoy their jobs. Examples include meaningful incentives proposed by hospitals, policy makers, and payers; a well-designed medical curriculum, including postgraduate education skills relevant to teaching; the wider availability of technologies; useful recommendations from peers; a rising number of evidence-based papers and guidelines; technologies that help save time and effort; and, generally, a good experience with e-patients [26-34] (see Textbox 1).

Among the many skills mentioned above, there are three cornerstones to this phenomenon that each e-physician should take into consideration: (1) the e-physician phenomenon means knowledge of, and positive attitude toward, digital technologies; (2) the e-physician phenomenon means the doctor-patient relationship will transition into a partnership; and (3) the e-physician phenomenon means that compassionate healing must remain the fundamental basis of health care.

**Table 1:** Summary of features of patient and medical professional empowerment.

Feature	Patient	Medical professional
Electronic	Uses digital technologies in their disease or health management	Uses digital technologies in their practice with ease
Equipped	Has digital health technologies at their disposal	Has digital health technologies at their disposal
Enabled	Enabled by their newly acquired access to information	Enabled by regulations and guidelines
Empowered	Empowered by the loss of the ivory tower	Empowered by technologies that support their job and e-patients
Engaged	Taking an active part in their care	Needs compassion and empathy to understand the feelings and points of view of patients, involving them throughout the whole healing process
Expert	Expert in the use of technologies in their care or health management	Expert in the use of technologies in their practice

**Summary of potential benefits and barriers of digital health adoption in medical practice, as well as potential actionable steps to address those barriers.**

Potential benefits for physicians of digital health adoption:

- Improves treatment efficiency
- Saves time
- Increases patient satisfaction
- Increases patient safety
- Improves diagnostic ability
- Improves daily productivity
- Improves physician-patient relationship and communication
- Improves interpersonal communication with colleagues
- Can ease the burden of routine check-ups for chronic patients
- Cost-savings for the medical practice

External and internal barriers to digital health adoption:

- Lack of time
- Increasing workload
- Lack of resources and financial difficulties
- Lack of reimbursements
- Lack of knowledge about digital health technologies
- Lack of trained medical staff
- Increase in misuse and misunderstanding of digital health technologies by patients
- A rise of overdiagnosis
- An increase in health disparities due to limited health literacy
- Increased administrative tasks
- Troubled patient data privacy and security
- Resistance from physicians (eg, losing control)
- A work culture refusing innovation

Potential actionable steps to address barriers to digital health adoption:

- Evidence-based digital health solutions
- Practice guidelines
- Availability of special training
- Support by colleagues and work environment
- Improving the quality, safety, and effectiveness of digital health technologies
- Facilitating laws and regulations for proper usage
- Recommendations from peers
- Perceived usefulness, or relative advantage, and compatibility (ie, with work process)
- Ease of use and user-friendly interfaces
- Incentive structures
- Patients’ positive attitudes and preferences regarding digital health solutions
- An innovation-oriented work culture

## The Reality Behind the Rise of E-Physicians

At the moment, factors that prevent the transition to digital health adoption in medical practice seem to be outweighing the positive elements. There are only a handful of examples where hospitals, policy makers, and payers offer good incentives to improve the use of meaningful technologies. There is a serious lack of guidelines and policies. Health care is a complex system; many disruptive technologies are still too expensive to become widely available. In addition, there is a general reluctance of peers to adopt digital health, and there are even patients who do not wish to become empowered. For the above-mentioned changes to occur, the following factors are indispensable: strengthening professional competence and reshaping the medical curriculum.

As KR Sethuraman stated, “The physicians of tomorrow are taught by the teachers of today using the curriculum of the past” [35]. Obviously, medical education must include preparation for the digital era with evidence-based examples of curricula, such as the course *Lessons in Digital Health* at Semmelweis Medical School [36]. This is an open-access course available worldwide and shows examples of physicians who are masters of using digital health technologies, but only to allow themselves more time to listen to patients discuss their health issues with undisturbed empathy.

There are already positive examples available, as illustrated below, about practicing physicians who embody the image of the e-physician that this paper describes:

1. Dr Wendy Sue Swanson advocates for the use of social media to strengthen communication between health care providers and patients. She supports the idea that technologies can assist patients and their families in becoming stewards of their own health. She also launched a company to help other physicians learn to use online tools more effectively in helping patients make informed decisions based on scientific evidence [37].
2. Dr Jay Parkinson is the founder a primary care practice that also uses online tools and platforms for remote care. He has been building services that explore what the Internet means to health care delivery [38].
3. Dr Bryan Vartabedian is considered one of health care’s most influential voices on social technology and medicine. He regularly expresses his views on patient-centricity, while also understanding medicine’s emerging digital culture and how new media can be leveraged by organizations and individual stakeholders [39].
4. Dr Bas Bloem, a Dutch professor of neurology and Director of the Parkinson Center in Nijmegen, advocates for placing patients at the center of disease and health management and is a popular voice advocating for the use of new technologies [40].

Such e-physicians could serve as role models for young students who aspire to practice medicine but are afraid of the burden of IT issues, time management, and a huge workload. The skills and approaches that characterize this era of e-physicians, such as face-to-face communication skills, digital literacy, interdisciplinarity, knowing where to find information, translating large amounts of data into insights for patients, among others, should always have been at the core of practicing medicine. However, the economical, technological, and administrative burden of the profession has not made it possible for most physicians to enjoy the benefits of their training, individual capabilities, and creativity. By understanding how digital health technologies can support or augment their capabilities, physicians would have the chance to practice the art of medicine like never before.

## Abbreviations

e-patient: empowered patient
e-physician: empowered physician
